# Closing the Gap between *the Inside* and *the Outside*: Interoceptive Sensitivity and Social Distances

**DOI:** 10.1371/journal.pone.0075758

**Published:** 2013-10-01

**Authors:** Francesca Ferri, Martina Ardizzi, Marianna Ambrosecchia, Vittorio Gallese

**Affiliations:** 1 Department of Neuroscience, University of Parma, Parma, Italy; 2 Brain Center for Social and Motor Cognition, Italian Institute of Technology (IIT), Parma, Italy; Royal Holloway, University of London, United Kingdom

## Abstract

Humans’ ability to represent their body state from within through interoception has been proposed to predict different aspects of human cognition and behaviour. We focused on the possible contribution of interoceptive sensitivity to social behaviour as mediated by adaptive modulation of autonomic response. We, thus, investigated whether interoceptive sensitivity to one's heartbeat predicts participants' autonomic response at different social distances. We measured respiratory sinus arrhythmia (RSA) during either a Social or a Non-social task. In the Social task each participant viewed an experimenter performing a caress-like movement at different distances from their hand. In the Non-social task a metal stick was moved at the same distances from the participant's hand. We found a positive association between interoceptive sensitivity and autonomic response only for the social setting. Moreover, only good heartbeat perceivers showed higher autonomic response 1) in the social compared to the non-social setting, 2) specifically, when the experimenter's hand was moving at boundary of their peripersonal space (20 cm from the participant's hand). Our findings suggest that interoceptive sensitivity might contribute to interindividual differences concerning social attitudes and interpersonal space representation via recruitment of different adaptive autonomic response strategies.

## Introduction

The integration of information about the internal bodily state and the external environment is crucial to adapt one's behaviour in social settings and everyday life. The ability to represent one's own internal body state is commonly referred to as interoception. Conceptualized as the sense of the physiological condition of the body [1], interoception has been hypothesized to have a primary role for basic homeostasis, behavioural motivations and interaction [2]. Empirical research on interoception has predominantly focused on a particular type of interoceptive sensitivity (i.e., sensitivity to stimuli originating inside of the body), that is, heartbeat perception. One reason is that there are only few bodily signals from the bodily “interior” that can be readily perceived (e.g. the heartbeat or signals from the guts), whereas the rest of internal activity is mostly “hidden” [3]. Individuals' sensitivity to their own heartbeat seems to be a trait-like characteristic. As such, it has been shown to interact with different aspects of human cognition and behaviour, for a review see [4]. For instance, it has been proposed that heartbeat detection sensitivity is relevant for emotional processing [5]–[7] and physiological reactivity towards emotional cues [8]. A recent study has demonstrated that the more accurately participants could track their heartbeat, the stronger the observed link between their heart rate reactions and their subjective arousal ratings of emotional images [8]. On the other hand, heartbeat detection sensitivity has been suggested to be a negative predictor of impairments in emotional awareness and regulation of emotions [9], [10]. More generally, interoceptive processes seem to contribute to the regulation of social behaviour. This is clearly manifest, for example, in the positive relation existing between individual interoceptive accuracy and social anxiety level [11]–[14].

Actual social settings typically require an individual to define the boundaries between oneself and the others. So far, however, only few studies have investigated interoceptive sensitivity as predictor of the representation of one's body and self-other boundaries [15], [16]. These studies showed that interoceptive sensitivity predicts the malleability of self-representations in response to multisensory integration. In particular, individuals with low interoceptive sensitivity experienced a stronger illusion of body ownership [15] and changes in self-other boundaries in response to multisensory stimulation [16]. However, in none of these studies participants were exposed to an actual social setting. In general, how interoceptive sensitivity impacts on engagement in social situations is a relevant and not yet well researched issue. An interesting question arising from previous empirical investigations [15]–[17] is, for instance, whether and what extent interoceptive sensitivity affects the representation of one's peripersonal space, as a multisensory-motor interface between body and environment, in social circumstances. The definition of peripersonal space [18], [19] originates from electrophysiological studies based on visual–tactile neurons identified in the premotor area F4 and the ventral intraparietal area (VIP) of the monkey brain [20]–[22]. The receptive fields of the VIP-F4 neurons are coded in somatic coordinates and anchored to various parts of the body. In particular, the visual receptive fields of F4 neurons around the hand extend from 5 to 35 cm from the tactile receptive fields [23]. Interestingly, Teneggi et al. [24] have recently demonstrated that peripersonal space representation is sensitive to social modulation, since its boundaries shrink when subjects face another individual, as compared to a mannequin, placed in far space.

In this work we aimed at investigating whether interoceptive sensitivity to one's heartbeat predicts modulation of participants' autonomic response to either social or non-social stimuli moving at different distances from the participant's body, that is, either in the far or in the near peripersonal space. We assumed that the ability to adapt to social environments does not merely depend on individual sensitivity to assess information from the external milieu, but also from within.

Participants viewed an unfamiliar hand performing a caress-like movement at different distances from their body. The experimenter exerting the caress-like movements was not visible to the participants. Despite this manipulation may appear quite artificial, it was chosen in order to avoid that the participants focused on experimenter's physical features (i.e., body size, face, eye gaze). We expected autonomic response to be induced as a function of interpersonal distance. This hypothesis was mainly based on our recent demonstration that expectation of being touched from a human hand, rather than the touch itself, can elicit participant's autonomic reactivity, provided that the approaching human hand entered the participant's peripersonal space [17].

Here we measured the respiratory sinus arrhythmia (RSA) as the dependent autonomic variable. RSA is one of the periodic components of heart rate variability, which tend to aggregate within several frequency bands [25]. RSA has been conceptualized as a phenomenon that directly results from the interaction between the cardiovascular and respiratory systems [26]. There is evidence suggesting that RSA response can be modulated by emotional processing [27], is positively correlated with social disposition [28] and can be considered as a marker for positive social functioning in children with autism [29], [30]. However, if interoceptive sensitivity predicts RSA response in social, compared to non-social, situations is a not yet researched issue.

In this study we firstly hypothesized that interoceptive sensitivity specifically predicts RSA in a social, compared to a non-social, setting. Then, given the assumption that information from the external environment and from within are integrated in the peripersonal space, we further hypothesized that the participant's interoceptive sensitivity might affect autonomic response towards social stimuli presented within or at the boundary of the participant's peripersonal space (i.e., as a function of social distance).

## Materials and Methods

### Participants

Twenty-four (11 males) right-handed [31] healthy volunteers (mean age 24±4, range  = 19–38) were selected for inclusion in the study. Individuals with either neurologic or cardiorespiratory or psychiatric diseases, as well as users of drugs interfering with the cardiac and respiratory activity, and heavy smokers (>25 cigarettes per day) [32] were excluded. As it is known that regular exercise influences autonomic tone, especially the vagal component [33], [34], which in turn is able to improve interoceptive awareness as assessed by heartbeat perception [35]–[37], only individuals not regularly involved in athletic or endurance sports were recruited. Moreover, as it is known that Body Mass Index (BMI) affects the ability to detect heartbeat sensations [38], [39], underweight (BMI<18.50 kg/m^2^) and obese (BMI>30.00 kg/m^2^) individuals, as defined by the “International Classification of adult underweight, overweight and obesity” of the World Health Organization [40], were not chosen for participation in the study.

All participants gave written informed consent and all experiments were conducted in accordance with the ethical standards of the 1964 Declaration of Helsinki. The experimental protocol was approved by the Ethical Committee of the University of Parma.

### Procedure

**Figure 1:**
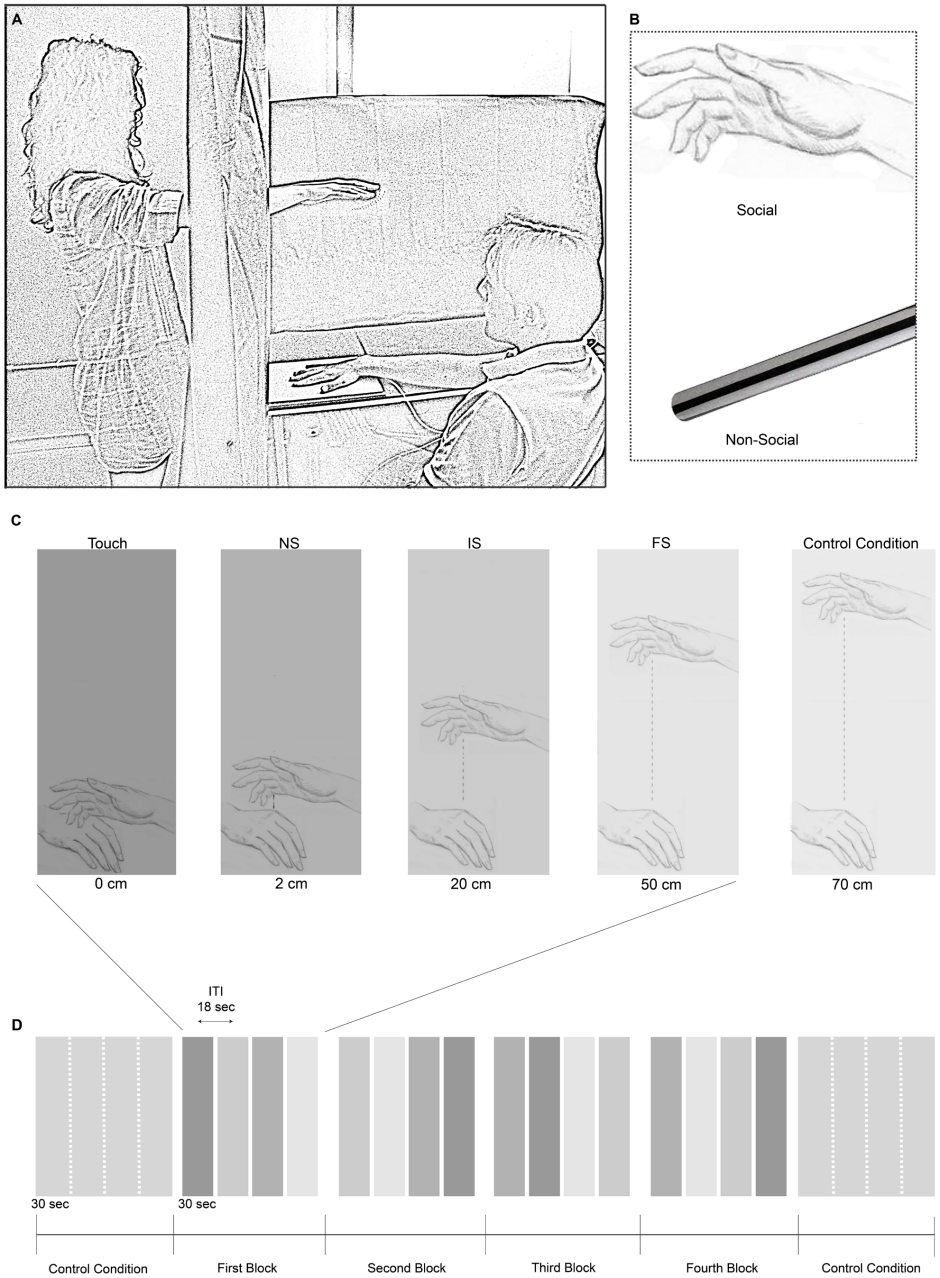
Social and Non-Social Task. (A) Experimental setup of the Social Task. (B) Examples of the experimental stimuli. (C) Schematic representation of experimental conditions: Touch, NS (Near-peripersonal Space), IS (Intermediate-Peripersonal Space), FS (Far-peripersonal Space) and Control Condition. (D) Experimental design of the Social and Non-Social Task.

The study consisted of two experimental sessions. In preparation for each session, participants were required to abstain from alcohol, caffeine and tobacco for 2 hours prior to each session [41]. After arrival at the laboratory for the first session participants were asked to fill in the following questionnaires: the Beck Depression Inventory (BDI) [42], the State-Trait Anxiety Inventory (STAI) [43] and the Autism-spectrum quotient (AQ) [44], assessing their depressive tendencies, anxiety and autistic traits, respectively. Then, they were asked to perform one of the two tasks described below: A) the Social Task, B) the Non-Social Task (Figure 1, A–B). Each participant performed the tasks in two separate experimental sessions taking place in different days. In each session participants were led into a quiet and soft illuminated room and were fitted with Ag-AgCl adhesive disposable electrodes for electrocardiogram (ECG). All recordings were performed in the same room with participants instructed to relax and to remain as still as possible during recording to minimize motion artefacts. At the beginning of the experimental session a 2-minute resting baseline ECG recording was done, in which participants were instructed to simply sit quietly with their eyes open. Subsequently, participants were administered with one of the two tasks described below (subsections A and B; see also Figure 1, A–B). Moreover, in the first experimental session, after a pause all participants completed the heartbeat perception task (see subsection C). All measurements were done in a comfortable sitting position of the participants.

#### A) Social Task

Participants were asked to sit in a comfortable relaxed position, right arm placed in a fixed location on the table in front of them. An experimenter stood at the participant's left side, hidden behind a black curtain (Figure 1, A). The experimenter was of opposite gender than the participant. She/he moved her/his hand simulating a caressing movement (1 Hz) at different distances from the participant's hand, according to the experimental condition (see below and Figure 1, C). The experimenter followed audio instructions delivered via earphones to perform controlled movements. The duration of simulation of caressing was kept fixed at 30 sec in each trial, at the end of which the experimenter withdrew her/his hand behind the curtain. The intertrial interval was 18 sec. Experimental conditions were the following: 1) Touch; 2) Near-peripersonal Space (NS, 2 cm from the participant's hand); 3) Intermediate-peripersonal Space (IS, 20 cm from the participant's hand); 4) Far-peripersonal Space (FS, 50 cm from the participant's hand). In a Control condition of the Social Task (Social Control condition, SC) the experimenter's hand moved at 70 cm from the participant's hand. We selected these distances according to previous neuropsychological studies [45], [46] and research on multisensory representations of peripersonal space [47], [48]. Specifically, the spatial distance for the Control condition was chosen on the basis of Holmes and Spence's [48] proposal, also supported by recent neuroimaging studies [49], that the limits of peripersonal space could be up to 70 cm in humans depending on the body part. Each experimental condition (Touch, NS, IS, FS) was presented once in a block, in random order. The duration of each block was 192 sec. The experiment consisted of four blocks. SC condition was presented before (2 min) the first and after (2 min) the last block (Figure 1, D). Participants were asked to feel comfortable and to carefully follow with their gaze what would have occurred within the space surrounding their hand.

At the end of the Social Task participants were asked to rate the comfort of each condition, using a 101-point visual analogue scale (VAS), with 0 corresponding to very little and 100 corresponding to very much. Participants were required to respond “How much they felt good when the hand was there during the task”, being the position simultaneously showed by the experimenter's hand, in random order. Thus, participants were not provided with any explicit indication of distances. During the subjective rating of comfort, participant's right hand was in the same position as during the task.

#### B) Non-Social Task

Experimental procedure and conditions (Touch, NS, IS, FS) were the same as in the Social Task except for the fact that participants viewed an object (a metal stick), instead of a human hand (Figure 1, B), coming out from the black curtain and simulating a caressing movement. The metal stick was moved by an invisible experimenter at the same frequency (1 Hz) and distances from the participant's hand (0, 2, 20, 50 cm) as in the Social Task. Similarly, the experimenter could keep the timing of inanimate caress-like movements under control following audio instructions delivered via earphones. As in the Social Task, a Control condition was presented at the beginning (2 min) and at the end (2 min) of the experiment. In the Non-Social Control condition (NC), the metal stick was moved at 70 cm from the participant's hand. At the end of the Non-Social Task, participants rated the comfort of each Non-Social condition following the same procedure as for rating of Social conditions (see above).

#### C) Heart beat monitoring task

Heartbeat perception was measured using the Mental Tracking Method [50] that has been widely used to assess interoceptive awareness, has good test–retest reliability (up to.81) [14], [51] and highly correlates with other heartbeat detection tasks [52]. Participants were instructed to start silently counting their own heartbeat on an audiovisual start cue until they received an audiovisual stop cue. After one brief training session (15 s), the actual experiment started. This consisted of four different time intervals of 100 s, 45 s, 35 s and 25 s, presented in random order across participants. Participants were asked to tell a second experimenter the number of heartbeats counted at the end of each interval. Throughout, participants were not permitted to take their pulse, and no feedback on the length of the counting phases or the quality of their performance was given. Heartbeat perception score was first calculated as the mean score of four heartbeat perception intervals according to the following transformation [50], [53]:
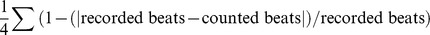



According to this transformation, heartbeat perception score can vary between 0 and 1, with higher scores indicating small differences between recorded and counted heartbeats (i.e., higher interoceptive sensitivity).

The median value of interoceptive sensitivity was 0.65 (SD = 0.14). Distribution of perception scores was tested for normality by using the Shapiro-Wilk test (p = 0.46). Despite it is known that the median can differ from study to study depending on the instructions used [54], this value was consistent with previous literature on the heartbeat tracking task [5], [39] and also with the median (0.65; SD = 0.17) calculated on 81healthy volunteers (45 males; mean age  = 24.14, SD = 4.1, ranging from 18–36 years) recruited for different studies on the heartbeat tracking task conducted in our lab. Then, using a median split method [15], [55], the group of 24 participants were split into two groups of high interoceptive sensitivity (High interoception Group, HG) and low interoceptive sensitivity (Low interoception Group, LG; see Table 1).

**Table 1:** Descriptive statistics.

	N.	Age (years)	Gender(male)	Cardiac IS	BMI (Kg/m^2^)	Traininig (hours per week)	BDI	STAI - Y2	AQ
**Total**	24	24.12±3.91	11	0.63±0.14	23.42±3.31	2.72±2.86	6.21±6.72	40.08±10.85	107.83±14.33
**HG**	12	24.33±4.94	5	0.74±0.06	23.67±3.61	2.40±2.38	5.41±5.80	41.41±8.32	102.58±9.56
**LG**	12	23.91±2.74	6	0.51±0.10	23.16±3.12	3.04±3.34	7.00±7.71	38.75±13.15	113.08±16.67

Mean values ± standard deviations for the Total Sample (Total), the High interoception Group (HG) and the Low interoception (LG) group. IS =  Interoceptive Sensitivity; BMI =  Body Mass Index; STAI – Y2 =  State Trait Anxiety Inventory; BDI =  Beck Depression Inventory; AQ =  Autism Quotient.

### Electrocardiogram (ECG) and Respiratory Sinus Arrhythmia (RSA) response

Three Ag/AgCl pre-gelled electrodes (ADInstruments, UK) with a contact area of 10 mm diameter were placed on the wrists of the participants in a Einthoven's triangle configuration to monitor ECG (Powerlab and OctalBioAmp 8/30, ADInstruments, UK).

The ECG was sampled at 1 KHz and online filtered by the Mains Filter with negligible distorting effect on ECG waveforms. The peak of the R-wave of the ECG was detected from each sequential heartbeat and the R-R interval was timed to the nearest msec. The R-R intervals were edited. Editing consisted of a software artefacts detection (artefacts threshold 300 msec) followed by a visual inspection of the ECG recorded signal. Artefacts were then edited by integer division or summation. The amplitude of Respiratory Sinus Arrhythmia (RSA) was quantified with CMetX (available from http://apsychoserver.psych.arizona.edu) [56]. This approach is basically a time-domain method but, like spectral techniques, allows derivation of components of heart rate variability within specified frequency bands [25]. The amplitude of RSA was assessed as the variance of heart rate activity across the band of frequencies associated with spontaneous respiration. RSA estimates were calculated using the following procedures [56]: a) linear interpolation at 10 Hz sampling rate; b) application of a 241-point FIR filter with a 0.12–0.40 Hz bandpass; c) extraction of the band passed variance; d) transformation of the variance in its natural logarithm. According to guidelines [25], these procedures were applied to epochs of 30 sec, corresponding to the duration of each experimental trial. Then, RSA values corresponding to Touch, NS, IS and FS conditions in each task were separately computed as the average of four 30 sec - epochs. Consistently, RSA values corresponding to SC and NC conditions were computed as the average of the last two 30 sec - epochs recorded before the first block and the first two 30 sec - epochs recorded after the last block of either the Social task or the Non-Social task, respectively. Similarly, baseline RSA values were computed as the mean of four 30 sec – consecutive epochs. RSA response to Touch, NS, IS, FS conditions and to the control condition were then separately obtained for the Social task and for the Non-Social task as changes from resting baseline RSA values to reactivity during each condition and each task. Heart rate data were used for assessing the heartbeat perception score.

### Questionnaire data

Since there is evidence suggesting that depression symptoms and RSA interact [57], [58], participants were required to fill in the Italian version of the BDI [59]. The BDI [42] is a widely 21-item multiple-choice self-report inventory that measures the presence and severity of affective, cognitive, motivational, psychomotor, and vegetative symptoms of depression. Each question has a set of at least four possible answer choices regarding how the subject has been feeling in the last week. Higher total scores indicate more severe depressive symptoms. Similarly, as it has been shown that anxiety interacts with RSA [60]–[62] and also because there is evidence suggesting a positive association between cardiac awareness and anxiety [14], [36], [63], [64], the participants filled in the Italian version of the STAI [65]. The STAI [43] is a 40 item scale, which assesses both state and trait anxiety and represents well-validated and reliable self-report measures of dispositional and state anxiety. Respondents are asked to indicate to what degree each item describes their dispositional and situational feelings on a four-point Likert-type scale (where 1 =  “not at all” and 4 =  “very much so”). Finally, since lower amplitude RSA and faster heart rate has been proposed to be associated with autism [29], [30], the participants filled in the Italian version of the AQ [66]. The Autism Spectrum Quotient [44] is a self-administered, 50 items forced-choice questionnaire for evaluate the presence of autistic traits across five domains (social skill, attention switching, attention to detail, communication and imagination) in both clinical and non-clinical samples. Respondents are asked to indicate how much they agree with each item (“definitely agree”, “slightly agree”, “slightly disagree” or “definitely disagree”).

### Data analysis

Pearson correlations were calculated between the heartbeat perception score and the RSA response to either the Social or the Non-social Control conditions (i.e., changes from baseline RSA values to reactivity during SC and NC, respectively) to investigate whether and to what extent heartbeat perception sensitivity predicts RSA response either in a social or in a non-social situation, or both. In order to analyze if the association between heartbeat perception score and RSA response was mediated by Age, Gender, Body mass, Anxiety, Autistic traits and depression tendencies, Age, Gender, BMI, STAI score, AQ score and BDI score were included as predictors in hierarchical regression analyses (forward stepping) with RSA response to either SC condition or NC condition as criterion and heartbeat perception score as a predictor. Differences between good and poor heartbeat perceivers regarding autonomic reactivity to SC and NC conditions were further confirmed by means of repeated measures ANOVA with “group”(High, Low) as between-subjects factor and “context” (Social, Non-social) as within-subjects factor. The Fisher test was used for all post-hoc comparisons.

Then, we wanted to investigate whether and to what extent heartbeat perception sensitivity predicts RSA response in a social situation, compared to a non-social situation, as a function of different peripersonal space distances. First, for each task (the Social Task and the Non-Social Task) RSA responses to the NS, IS and FS conditions were normalized on the RSA response to the Touch condition, in order to get rid of individual variability associated to the experience of touch per se, thus keeping only the modulation associated to the approaching of a human hand or an object in the peripersonal space.

Indeed, it is known that anticipation of a sensory stimulus and processing of the somatosensory stimulus itself engage similar brain [67], [68] and autonomic [17] activities. Then, Pearson correlations between heartbeat perception scores and normalized RSA response to NS, IS and FS in each task were performed to assess whether interoceptive sensitivity predicts autonomic reactivity in a social situation, compared to a non-social situation, as a function of different peripersonal space distances. To account for multiple comparisons, we used Bonferroni correction and considered significant only the correlation for which p<0.017 (i.e., p value / total number of comparisons, 0.05/3). Differences between good and poor heartbeat perceivers regarding autonomic reactivity in a social situation vs. a non-social situation, as a function of different peripersonal space distances were further confirmed by means of repeated measures ANOVA with “group”(High, Low) as between-subjects factor, Task (Social, Non-Social) and Distance (Touch, NS, MS, FS), as the within-subject factors. The Fisher test was used for all post-hoc comparisons. Finally, Pearson correlations between heartbeat perception scores and comfort ratings of Touch, NS, IS and FS conditions were performed to see whether interoceptive sensitivity predicts also the explicit appreciation of the presence of another's hand at different peripersonal space distances. To account for multiple comparisons, we used Bonferroni correction and considered significant only the correlation for which p<0.012 (i.e., p value / total number of comparisons, 0.05/4). Differences in subjective ratings of comfort during Social and Non-Social Task in the HG and LG were investigated by means of repeated measures ANOVA with “group”(High, Low) as between-subjects factor and “context” (Social, Non-social) and “distance” (Touch, NS, IS, FS) as within-subjects factors. The Fisher test was used for all post-hoc comparisons.

## Results

### Interoceptive awareness and RSA response to Social and Non-social control conditions

**Figure 2:**
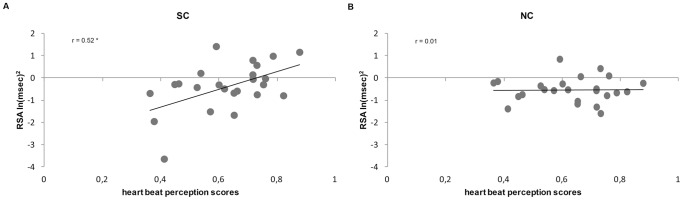
Correlation between heartbeat perception score and RSA response to Social and Non-Social Control conditions. (A) Correlation between heartbeat perception score and RSA response to the Social Control condition for all participants. (B) Correlation between heart beat perception scores and RSA response to the Non-Social Control condition for all participants. SC =  Social Control condition; NC =  Non-Social Control condition. * p<0.05.

Mean heartbeat perception score for all participants (N = 24) was M = 0.63, SD = 0.14 ranging from 0.36 to 0.88. Mean RSA response to SC was M = 0.40 ln(msec)^2^, SD = 1.08 ranging from −3.66 to 1.40. Moreover, mean RSA response to NC condition was M = −0.54 ln(msec)^2^, SD = 0.56 ranging from −1.61 to 0.82 (see Table 1 for further details). There was a significant positive correlation between the heartbeat perception scores and RSA response to SC condition (r_24_ = 0.52, p<0.005, two-tailed), whereas no significant correlation was found between the heartbeat perception scores and RSA response to NC condition (r_24_ = 0.01, p = 0.96, two-tailed) (Figure 2). Accordingly, hierarchical regression analyses (forward stepping) demonstrated that the criterion RSA response to SC condition was explained by the heartbeat perception score (t = 2.86, β = 0.52, p<0.01) with a total of 24% explained variance for the regression model (F_(1,22)_ = 8.19, p<0.01, R = 0.52, R^2^ = 0.27). All other predictors were not included in the regression model. Differently, when hierarchical regression analysis (forward stepping) with RSA response to NC condition as criterion and with heart beat perception score, Age, Gender, BMI, STAI score, AQ score and BDI score as predictors was performed, no predictors were included in the regression model.

### RSA response of High and Low interoception Groups to Social and Non-social Context

**Figure 3:**
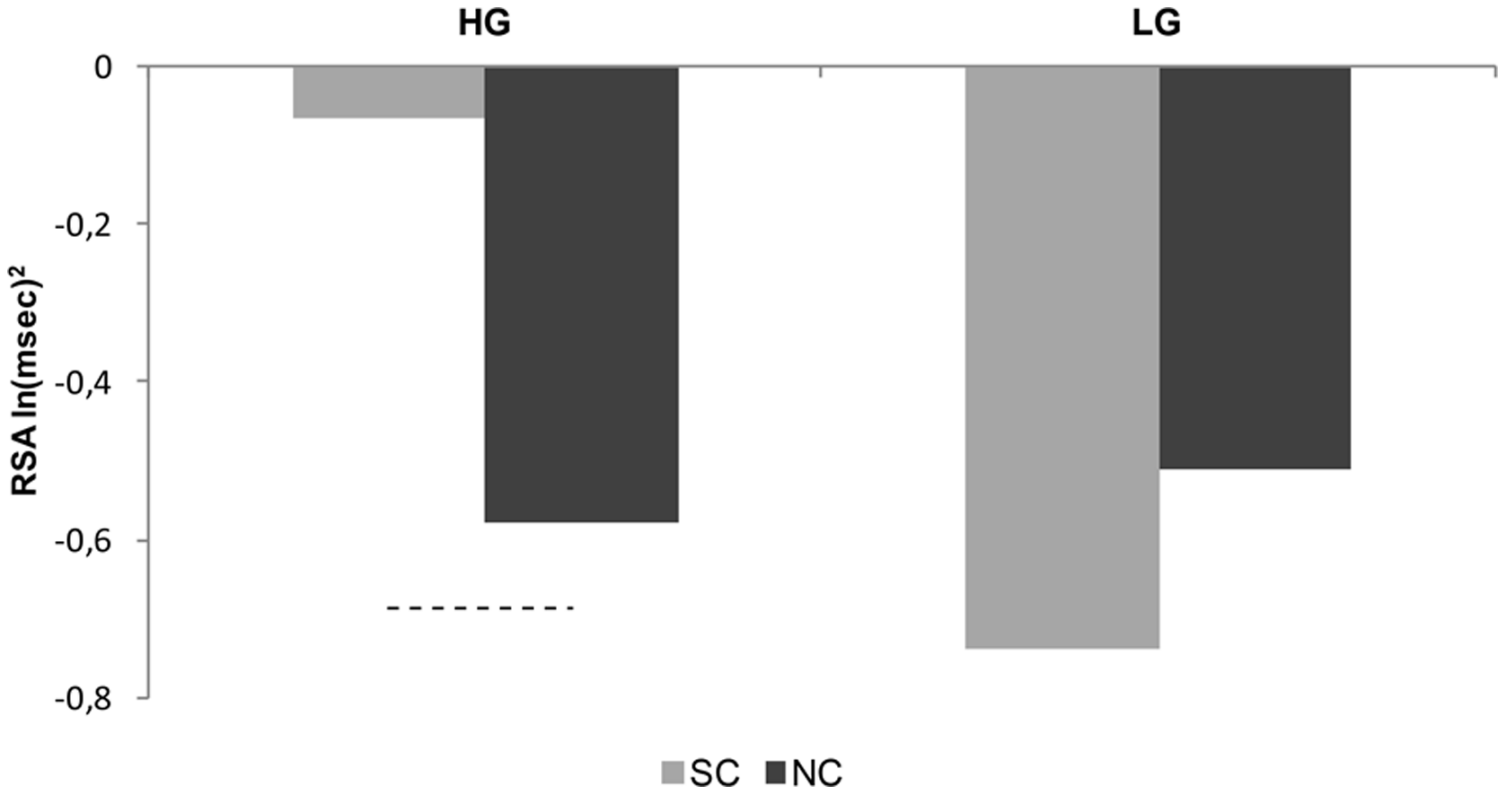
RSA response of High and Low interoception Groups to Social and Non-social Control conditions. HG =  High interoception Group; LG =  Low interoception Group; SC =  Social Control condition; NC =  Non-Social Control condition. Dashed line indicates p<0.05. See Table 2 for standard deviations.

Mean heartbeat perception score for the HG (N = 12, 5 males) was M = 0.74, SD = 0.06. Mean heartbeat perception score for the LG (N = 12, 6 males) was M = 0.51, SD = 0.10 (see Table 1 for further details). Confirming the results described above, ANOVA showed a significant Context by Group interaction (F_1,22_ = 4.17, p = 0.05, η^2^_p_ = 0.16), because RSA responses to social and non-social context were significantly different in the HG [SC: M = −0.06 ln(msec)^2^, SD = 0.83; NC: M = −0.58 ln(msec)^2^, SD = 0.59; p = 0.05], but not in the LG [SC: M = −0.73 ln(msec)^2^, SD = 1.23; NC: M = −0.51 ln(msec)^2^, SD = 0.56; p = 0.38] (Figure 3).

### Correlation between heart beat perception scores and RSA response at different distances

**Figure 4:**
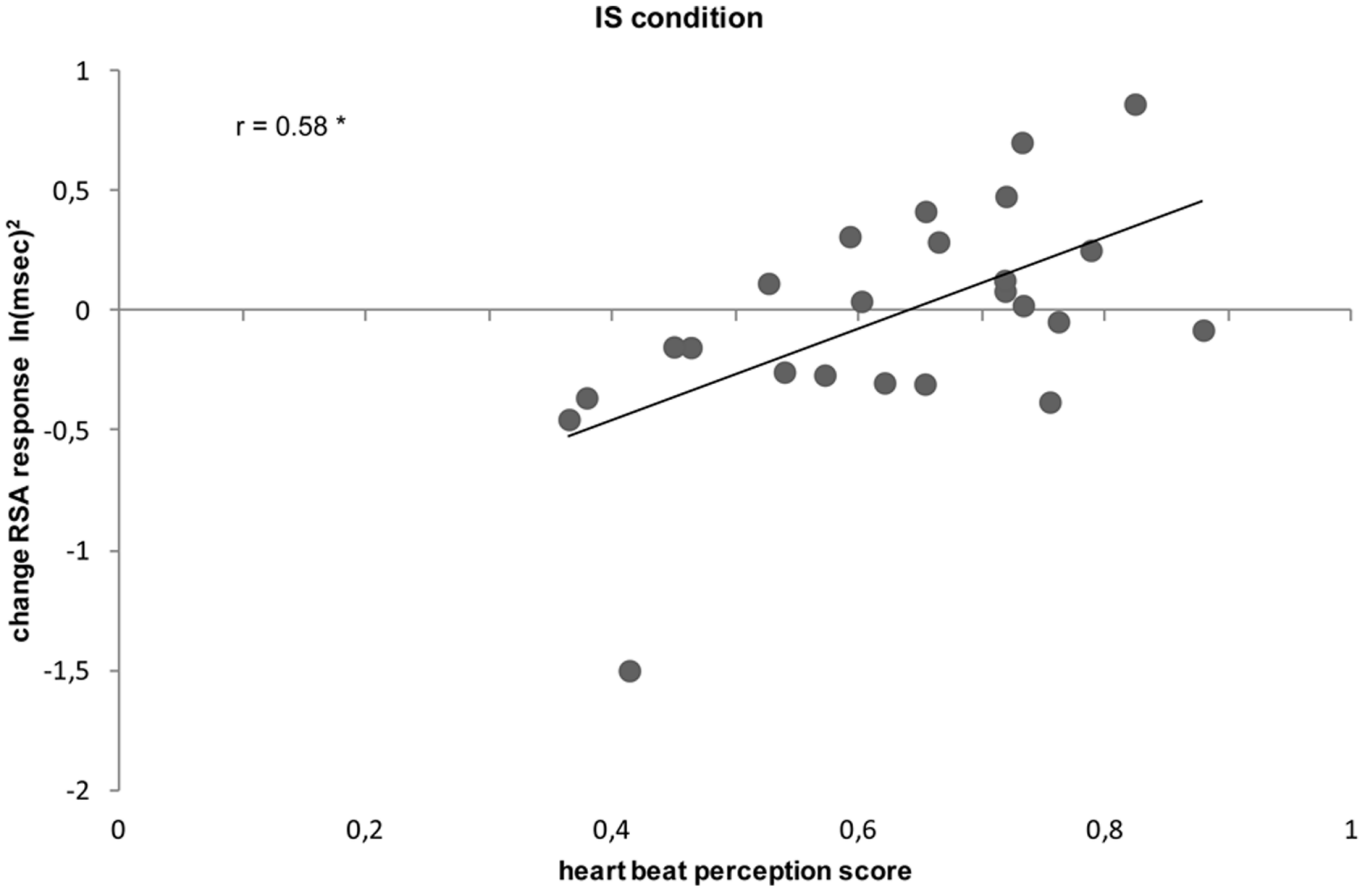
Correlation between heartbeat perception score and RSA response to IS condition. Correlation plot between heartbeat perception scores and change in RSA response from the Touch to the IS condition in the social context. IS =  Intermediate-Peripersonal Space. * p<0.05.

For the Social Task, a significant positive correlation was find only between heartbeat perception score and change in RSA response from the Touch to the IS condition (r_24_ = 0.58, p<0.05, two-tailed; Figure 4). Differently, for the Non-Social Task no significant correlation was found between heartbeat perception score and change in RSA response from the Touch condition to any other experimental condition (all p_s_>0.05).

### RSA response of High and Low interoception Groups as a function of distances

**Figure 5:**
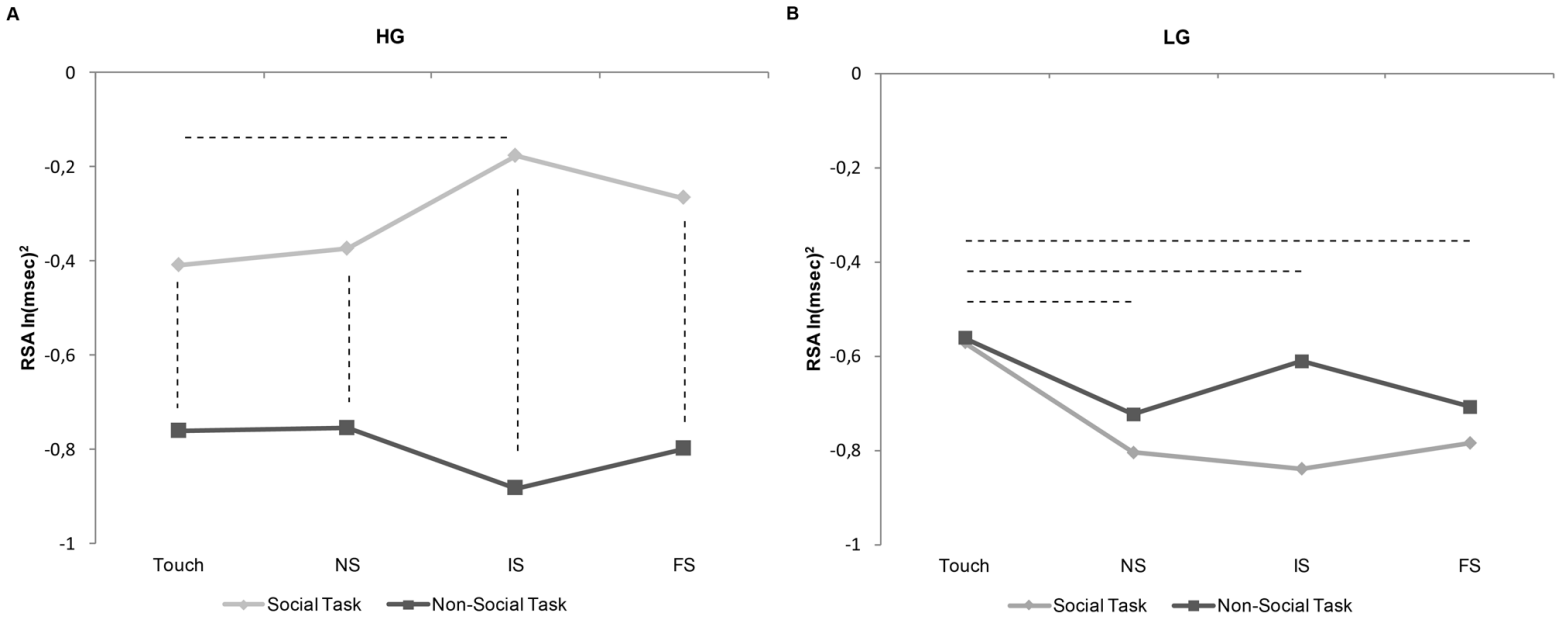
RSA response of High and Low interoception Groups as a function of distances in Social and Non-Social Task. (A) RSA response of the High interoception Group to each experimental condition in the Social (light gray line) and the Non-Social Task (dark gray line). (B) RSA response of the Low interoception Group to each experimental conditions in the Social (light gray line) and the Non-Social Task (dark gray line). NS =  Near-peripersonal Space; IS =  Intermediate-Peripersonal Space; FS =  Far-peripersonal Space; HG =  High interoception Group; LG =  Low interoception Group. Dashed line indicates p<0.05. See Table 2 for standard deviations.

RSA response at the different experimental conditions are reported in Table 2 for both HG and LG. Confirming the results obtained from the correlation analysis described above, ANOVA showed a significant 3-way interaction Group by Task by Distance (F_3,66_ = 3.02, p<0.05, η^2^_p_ = 0.12) (Figure 5). Post hoc comparisons demonstrated that RSA response in the HG was higher in the Social Task than in the Non-Social Task at each distance (Touch: M = −0.40 ln(msec)^2^, SD = 0.98 vs. M = −0.76 ln(msec)^2^, SD = 0.74; NS: M = −0.37 ln(msec)^2^, SD 0.95 vs. M = −0.75 ln(msec)^2^, SD = 0.86; IS: M = −0.18 ln(msec)^2^; SD = 0.90 vs. M = −0.88 ln(msec)^2^, SD = 0.86; FS: M = −0.27 ln(msec)^2^, SD = 1.00 vs M = −0.80 ln(msec)^2^, SD = 0.75; all p_s_<0.05). Moreover, RSA response of the HG in the Social Task was significantly higher for the IS condition than the Touch condition [MS: M = −0.18 ln(msec)^2^, SD = 0.90; Touch: M = −0.40 ln(msec)^2^, SD = 0.98; p<0.05]. Finally, RSA response of the LG in the Social task was significantly higher for the Touch condition than all the other conditions (Touch: M = −0.57 ln(msec)^2^, SD = 1.04; NS: M = −0.80 ln(msec)^2^, SD = 1.42; IS: M = −0.84 ln(msec)^2^, SD = 1.42; FS: M = −0.78 ln(msec)^2^; SD = 1.33; all p_s_<0.05] (see Table 2).

**Table 2:** Cardiac parameters and Subjective rating of Comfort in the Social and the Non-Social Task.

	High interoception Group		Low interoception Group	
**RSA - ln(msec)^2^**	**Social Task**	**Non-Social Task**	**Social Task**	**Non-Social Task**
*Touch*	−0.40±0.98	−0.76±0.74	−0.57±1.04	−0.56±0.57
*NS*	−0.37±0.95	−0.75±0.86	−0.80±1.42	−0.72±0.69
*IS*	−0.18±0.90	−0.88±0.86	−0.84±1.42	−0.61±0.49
*FS*	−0.27±1.00	−0.80±0.75	−0.78±1.33	−0.71±0.63
*Control Condition*	−0.06±0.83	−0.58±0.59	−0.73±1.23	−0.51±0.56
**HR - bpm**				
*Touch*	−2.45±11.57	−2.87±3.35	−0.23±11.43	−2.86±3.26
*NS*	−3.04±10.38	−2.14±3.76	1.33±12.70	−1.34±3.01
*IS*	−1.56±10.83	−1.15±3.29	2.22±12.30	−0.80±1.93
*FS*	−0.76±10.54	−0.58±3.47	2.00±12.03	−0.49±3.18
*Control Condition*	−1.93±9.70	−0.77±3.11	2.27±11.58	−0.14±2.41
**Rating of comfort - %**				
*Touch*	52.08±35.53	49.50±33.19	54.33±40.27	33.50±33.65
*NS*	52.00±31.25	48.00±24.70	54.45±33.00	30.50±27.38
*IS*	65.50±26.13	56.17±19.71	58.41±22.67	36.58±20.94
*FS*	72.00±23.07	53.67±21.75	56.75±24.31	45.83±16.59
*Control Condition*	68.08±30.62	64.75±22.00	53. 92±28.42	64.00±20.27

Mean values ± standard deviations of cardiac parameters (RSA =  Respiratory Sinus Arrhytmia and HR =  Heart Rate) and Subjective rating of Comfort for the High and the Low interoception Groups. RSA and HR are reported as changes from the resting baseline values. NS =  Near-peripersonal Space; IS =  Intermediate-Peripersonal Space; FS =  Far-peripersonal Space; bpm =  beats per minute.

### Analyses of comfort ratings

When Pearson correlations between heartbeat perception scores and comfort ratings of Touch, NS, IS and FS conditions in Social context were performed no significant correlation was found in both groups (all p_s_>0.05) (See Table 2). ANOVA showed a significant Context effect (F_1,22_ = 6.12, p<0.05, η^2^_p_ = 0.21). Post hoc comparisons demonstrated that all participants showed higher comfort rating for the Social context (M = 58.76%, SD = 29.67) than for the Non-Social context (M = 48.25%, SD = 26.27) (all p_s_<0.05). No significant differences were found between the two groups and among experimental distances (See Table 2).

## Discussion

In this work we investigated, for the first time, whether interoceptive sensitivity to one's heartbeat predicts modulation of participants' autonomic response at different social distances. We started from the idea that human ability to adapt to complex social settings does not merely reflect high sensitivity in assessing information from the external milieu, but also from *the inside*. Indeed, both interoceptive sensitivity [4] and social skills [69] have been proposed to be exceptionally, despite not specifically, developed features in humans, through which they attend upon body's homeostatic needs [1], [2], [70], [71]. This study looked at a possible relation between them and revealed a role of interoceptive sensitivity in shaping social behaviour by means of recruiting different autonomic response strategies. Adaptive autonomic responses, and interoceptive sensitivity, likely play a critical role in a situation in which, for example, perceived threat from others in peripersonal space is the most salient factor in mediating equilibrium between interpersonal distance and social interaction [72], [73]. Accordingly, it has been previously suggested that the development of social skills in humans is tightly related to our autonomic response strategies [74], [75].

We assessed interoceptive sensitivity through the heartbeat perception task. Here it is due to note that there is up to now only few studies showing that interoceptive sensitivity as measured by heartbeat perception is related to interoceptive sensitivity for other bodily internal signals belonging to other organ systems [3], [76]. Moreover, participants' autonomic response in social and non-social settings was assessed by measures of RSA, conceptualized as an index of self-regulation and social engagement [28], [74], [75], [77], [78]. Specifically, the higher the RSA amplitude the higher the social disposition. Accordingly, higher RSA amplitude at baseline has been proposed to be a marker for positive social functioning in children with autism [29], [30]. From the physiological perspective, RSA is a cardiorespiratory phenomenon characterized in mammals by heart rate (HR) or R-R interval (RRI) fluctuations that are in phase with inhalation and exhalation. RSA is frequently employed as an indicator of cardiac vagal tone ([79], but see also [26]). Dynamic changes in RSA may occur across a wide variety of physiological, behavioral and psychological conditions. For example, during continuous mental processing (e.g. a cognitive reaction-time task) RSA may be lower than during quiet relaxation, when breathing is slower and deeper [80]. In particular, there is evidence that RSA is significantly reduced during tasks requiring sustained attention [81], [82], which is consistent with the negative values we obtained as changes from baseline RSA to reactivity for all conditions in both tasks. Another possible explanation is that, despite in normal context a caress-like movement could be expected to increase relaxation (i.e., increase parasympathetic activity), in a situation in which a covered and unfamiliar person exerts these movements, like in our experimental settings, this might have contrary effects. If present, such effects would impinge on all conditions in both Social and Non-social tasks. That would hardly be consistent with the results of the comfort rating showing that Social conditions were rated higher than Non-social conditions by both the High and the Low interoception groups.

As the first interesting result, we found a positive relation between interoceptive sensitivity and RSA response specifically for the social setting. Indeed, only participants scoring higher at the heartbeat detection task showed higher RSA values (i.e., less RSA reduction compared to baseline) for Social Control condition, but not for Non-Social Control condition. This result might suggest that in contexts affording social interactions people with higher or lower interoceptive sensitivity specifically diverge in their autonomic response strategy. Indeed, good heartbeat perceivers seem to be characterized by higher social disposition (higher RSA; [28], [74], [75], [77], [78]). One may argue that higher RSA could also reflect effortful emotion regulation in presence of a moderately stressful stimulus, caused by a moderate level of unpleasantness in the given situation or by social anxiety. These alternative hypotheses can be both excluded based on our results. On the one hand, the comfort rating results showed that both groups experienced the social setting as more pleasant than the non-social. On the other hand, regression analysis revealed that participants' anxiety did not significantly contribute to the association between interoceptive sensitivity and RSA response.

Second, when we tested whether interoceptive sensitivity predicts RSA response at different social and non-social distances after subtracting possible effects due to expected/anticipated touch, we found a positive correlation only for the social setting between heartbeat perception scores and RSA response at the IS distance (20 cm from the participant's hand). ANOVA confirmed this results. Indeed, it showed that good heartbeat perceivers, but not poor heartbeat perceivers, responded higher in the social compared to the non-social settings at each distance. Again, this suggests that good heartbeat perceivers are generally characterized by higher social disposition. Moreover, in the Social task RSA response of the High interoception Group was significantly higher for the IS condition than the Touch condition, suggesting that their autonomic strategy to engage in social interaction likely requires effortful emotion regulation as soon as another enters one's peripersonal space. This result is consistent with our previous observation that expectation of touch experience arising at the sight of a human hand approaching a rubber hand is enough to induce embodiment of the rubber hand only when the approaching stimulus (i.e. experimenter's hand) entered participants' peripersonal space (at a distance between 15 and 30 cm from the participant's hand) [17]. Finally, RSA response of the LG in the Social task was significantly higher for Touch condition than all the other conditions, suggesting that they have less efficient autonomic strategy to engage in social interaction. In other words, it seems that people with low interoceptive skills are also harder to engage in social interactions. They actually respond to the presence of another, provided that the other is very near to their own body. Based on previous literature on cardiac interoceptive sensitivity, at least two possible hypotheses can be formulated to elucidate these results. The first hypothesis refers to the evidence provided by Matthias and colleagues [83] that interoceptive awareness is positively related to the attentional processing of external visual stimuli. Thus, good heartbeat perceivers would be also more able to focus on behaviourally relevant information guiding adaptive strategies. The second hypothesis concerns the interaction between interoceptive sensitivity and body-representations [15]. High interoceptive sensitivity reduces the malleability of the multisensory representation of one' s body contributing to a more efficient processing of visuo-tactile body-related information occurring in close peripersonal space. As a consequence, interoceptive sensitivity might contribute to define safe social distances [84] and to judge the limits of a safe social space.

### Limitations and conclusion

Potential limitations of the present study could stem from the fact that RSA may reflect individual differences (e.g., novelty of/habituation to) in responding to experimental settings. Thus, reactivity to novelty, strangeness, funniness or any other aspect of the experimental situation may partially contribute to autonomic responses. However, we believe that this study can shed new light on the connection between interoceptive sensitivity and social interaction, suggesting that interoceptive sensitivity likely predicts inter-individual differences in recruiting adaptive autonomic response strategy in social settings and everyday life.
